# Divergent expression of claudin -1, -3, -4, -5 and -7 in developing human lung

**DOI:** 10.1186/1465-9921-11-59

**Published:** 2010-05-17

**Authors:** Riitta Kaarteenaho, Heta Merikallio, Siri Lehtonen, Terttu Harju, Ylermi Soini

**Affiliations:** 1 Institute of Diagnostics, Department of Pathology and Clinical Research Center , University of Oulu, Oulu, Finland; 2 Institute of Clinical Medicine, Department of Internal Medicine / Respiratory Research Unit, Centre of Excellence in Research and Clinical Research Center, University of Oulu and Oulu University Hospital, Oulu, Finland; 3 Clinical Research Center, and Institute of Clinical Medicine, Department of Surgery, University of Oulu and Oulu University Hospital, Oulu, Finland; 4 Department of Pathology and Forensic Medicine, School of Medicine, University of Eastern Finland, Kuopio, Finland

## Abstract

**Background:**

Claudins are the main components of tight junctions, structures which are associated with cell polarity and permeability. The aim of this study was to analyze the expression of claudins 1, 3, 4, 5, and 7 in developing human lung tissues from 12 to 40 weeks of gestation.

**Methods:**

47 cases were analyzed by immunohistochemisty for claudins 1, 3, 4, 5 and 7. 23 cases were also investigated by quantitative RT-PCR for claudin-1, -3 and -4.

**Results:**

Claudin-1 was expressed in epithelium of bronchi and large bronchioles from week 12 onwards but it was not detected in epithelium of developing alveoli. Claudin-3, -4 and -7 were strongly expressed in bronchial epithelium from week 12 to week 40, and they were also expressed in alveoli from week 16 to week 40. Claudin-5 was expressed strongly during all periods in endothelial cells. It was expressed also in epithelium of bronchi from week 12 to week 40, and in alveoli during the canalicular period. RT-PCR analyses revealed detectable amounts of RNAs for claudins 1, 3 and 4 in all cases studied.

**Conclusion:**

Claudin-1, -3, -4, -5, and -7 are expressed in developing human lung from week 12 to week 40 with distinct locations and in divergent quantities. The expression of claudin-1 was restricted to the bronchial epithelium, whereas claudin-3, -4 and -7 were positive also in alveolar epithelium as well as in the bronchial epithelium. All claudins studied are linked to the development of airways, whereas claudin-3, -4, -5 and -7, but not claudin-1, are involved in the development of acinus and the differentiation of alveolar epithelial cells.

## Introduction

During lung ontogenesis, the conducting airways are quite early lined by epithelium which consists of ciliated, secretory, intermediate and basal cells [1,2]. The epithelium of distal lung including type I and II pneumocytes and Clara cells lining the walls of respiratory bronchioles and alveoli, differentiate later than the cells of the conducting airways [3]. Epithelial cell differentiation is closely connected to the changes in the extracellular matrix and its proteins which are expressed in varying amounts and in distinct locations during human lung development [4,5].

Epithelial and endothelial cells interact with neighbouring cells through various kinds of cell-cell communication systems, such as tight, gap and adherens junctions. All types of junctions exist in lung epithelium, but knowledge of their development, exact function and distribution in the developing and adult human lung is incomplete. It was discovered less than a decade ago that the claudin family which nowadays contains 24 members, are proteins that make up the tight junctions [6]. Epithelial cells often express multiple claudin types, and they show a variable expression profile in different epithelia [7]. Similarly, expression of different claudins varies between different types of epithelial, endothelial and mesothelial tumors [8,9]. A mutation of claudin-16 is associated with a rare autosomal-recessive renal disorder, familial hypomagnesemia with hypercalciuria and nephrocalcinosis [10] and that of claudin-14 with deafness [11].

Expression of various claudins in the tissue of developing rat lung and in cultured fetal human lung cells has been previously documented [12,13]. Connexin 26, which is an element of the gap junctions, has been shown to be expressed in developing and adult human lung tissues [14]. Studies on the expression of claudins in different kinds of lung carcinomas and in human fibrotic lung disorders have been published [15-17]. Our previous study showed that in normal human adult lung, claudin-1, -2, -3, -4, -5 and -7 were expressed in the epithelium of bronchioles, whereas claudin-3, -4 and -7 were only located in type II pneumocytes in alveoli [18]. Claudin-5 is the only member of the claudin-family which is known to be expressed in endothelial cells [19]. Endothelial VE-cadherin at adherent junctions has been demonstrated to upregulate the gene encoding the tight junction adhesive protein claudin-5 [20]. So far there are no published studies on the expression of claudins in normal human developing lung at the tissue level.

Our aim was to study the expression and cell-specific localization of five different types of claudin, namely claudin -1, -3, -4, -5, and -7, in normal human developing lung at different gestational ages i.e. from week 12 to week 40 during the pseudoglandular, canalicular, saccular and alveolar periods. We hypothesized that the expression of the different claudins during ontogenesis of human lung might vary since they have distinct expression profiles in normal human lung.

## Materials and methods

### Patients and handling of specimens

Samples of lung tissue were retrieved from the files of the Department of Pathology, Oulu University Hospital. The study protocol was approved by the Ethical committee of the local hospital and the National Supervisory Authority for Welfare and Health. The study material for developing lung consisted of 47 cases of spontaneous abortion, stillbirth, and autopsied infants who had died for different reasons without lung disorders within 1-2 days after birth at the Oulu University Hospital between 1990 and 2002. Autopsies have been performed within the first day in most cases and within two days in 4 cases. The cause of death of the infants were abortion (n = 19), abruption of placenta (n = 9), rupture of fetal membranes (n = 2), feto-fetal transfusion (n = 2), sacral teratoma (n = 2), prolapse or aplasia of the umbilical artery (n = 3), placentitis or chorioamnionitis (n = 4), hemochromatosis (n = 1), hydrocephalus (n = 1), meningomyelocele (n = 1), encephalocele (n = 1), holoprosencephaly (n = 1) and hemorrhage of caput (n = 1). Infants with pneumonia, cardiac abnormalities or features of maceration were excluded. The gestational age of infants ranged from 12 to 40 weeks, corresponding to the pseudoglandular (day 52 to week 16, 13 cases), canalicular (weeks 16-28, 17 cases), saccular (weeks 28-36, 9 cases) and alveolar (weeks 36-40, 8 cases) periods. The clinical information was obtained from the patient records.

Lung samples, which had been taken from different parts of the left or right lung were fixed in 10% formalin and then dehydrated and embedded in paraffin. Sections 5 μm thick were stained with hematoxylin-eosin. All material was re-evaluated, and one representative tissue block from each case was selected for the immunohistochemical studies.

### Antibodies and immunohistochemical staining

The primary antibodies used for immunohistochemistry were all purchased from Zymed Laboratories Inc. (South San Fransisco, CA, USA) and designed to be used in formalin-fixed paraffin-embedded tissues. They were polyclonal rabbit anticlaudin 1 (clone JAY.8), polyclonal rabbit anticlaudin 3 (clone Z23.JM), monoclonal mouse anticlaudin 4 (clone 3E2C1), monoclonal mouse anticlaudin 5 (clone 4C3C2) and polyclonal rabbit anticlaudin 7 (clone ZMD.241). Before application of the primary antibodies, the sections were heated in a microwave oven in 10 mM citrate buffer, pH 6.0, for 10 min. After a 60-min. incubation with the primary antibody (dilution 1:50 for anticlaudins 1, 3, 4, 5 and 7), a biotinylated secondary anti-rabbit or anti-mouse antibody and the Histostain-SP kit (Zymed Laboratories) were used. In all the immunohistochemical evaluations, the colour was developed by diaminobenzidine and, subsequently, the sections were lightly counterstained with haemotoxylin and mounted with Eukitt (Kindler, Freiburg, Germany).

Negative controls were obtained by substituting non-immune rabbit or mouse serum and PBS for the primary antibodies.

### Scoring of the immunoreactivity

The extent and intensity of various claudins were evaluated semiquantitatively as negative (0), weak (+), moderate (++) or strong (+++) in different types of pulmonary cells, such as epithelial cells of bronchioles and bronchus, alveolar epithelium including pretype II cells, type I and II pneumocytes, endothelial cells, interstitial cells such as fibroblasts and myofibroblasts, and mesothelial cells. In the evaluation, membrane-bound positivity was considered as significant.

### Quantitative real-time reverse transcriptase polymerase chain reaction (RT-PCR)

In 23 cases representing all developmental periods (pseudoglandular, n = 4; canalicular, n = 8; saccular, n = 7; alveolar, n = 4) one tissue block was selected for quantitative real-time reverse transcriptase polymerase chain reaction (RT-PCR) analyses for claudin-1, -3 and -4. As it has been shown previously that RNA can isolated from paraffin embedded tissue material for expression profiling [21,22] the total RNA was isolated from five 10 μm thick slices from each sample with Purelink FFPE total RNA isolation kit according to the manufacturer's instructions (Invitrogen, Carlsbad, CA, USA). The isolated RNA was quantified and qualified spectrophotometrically and 0.5 μg of total RNA was converted to cDNA by RevertAid first strand cDNA synthesis kit (Fermentas, EU). Real Time PCR was performed with 5 μl cDNA template in a total volume of 25 μl, using the iQ5 Optical system (Bio-Rad Laboratories, Hercules, CA). The DNA- binding dye used in RT-PCR was SYBR Green I (iQ Custom SYBR green supermix, Bio-Rad Laboratories) which binds non-specifically to double-stranded DNA and the amount of bound dye was measured at each amplification cycle in a real time manner. RT-PCR reactions were performed in duplicate in 96-well plates. Cycling conditions were: at 95°C for three minutes, 40 cycles of amplification (each 95°C for 10 seconds and 56°C for 30 seconds), one minute at 95°C, one minute at 55°C and melt data acquisition. Primer sequences are shown in Table 1. The RT-PCR results of developing lung samples were compared to a lung sample of a normal human adult lung that was set to value 1 for each claudin separately. The relative quantities were calculated by Bio-Rad iQ5 standard edition optical system software version 2.0 by ΔΔC_T _method [23]. GAPDH was considered as a reference gene and a sample of normal adult lung as a reference sample. The mean values and standard deviations were calculated for pseudoglandular, canalicular, saccular and alveolar periods and the results were analysed statistically by Kruskal Wallis test.

**Table 1:** Sequences of primers used for qRT-PCR.

*Target*	*Reverse primer (5'-3')*	*Forward primer (5'-3')*
***GAPDH***	GAGTCAACGGATTTGGTCGT	GACAAGCTTCCCGTTCTCAG
***claudin 1***	CCGGCGACAACATCGTGAC	CGGGTTGCTTGCAATGTGC
***claudin 3***	CGCGAGAAGAAGTACACGG	CCTTAGACGTAGTCCTTGCGG
***claudin 4***	CGCATCAGGACTGGCTTTATCTC	CAGCGCGATGCCCATTA

## Results

### Immunohistochemistry

#### Pseudoglandular period (weeks 12 to 16)

**Figure 1:**
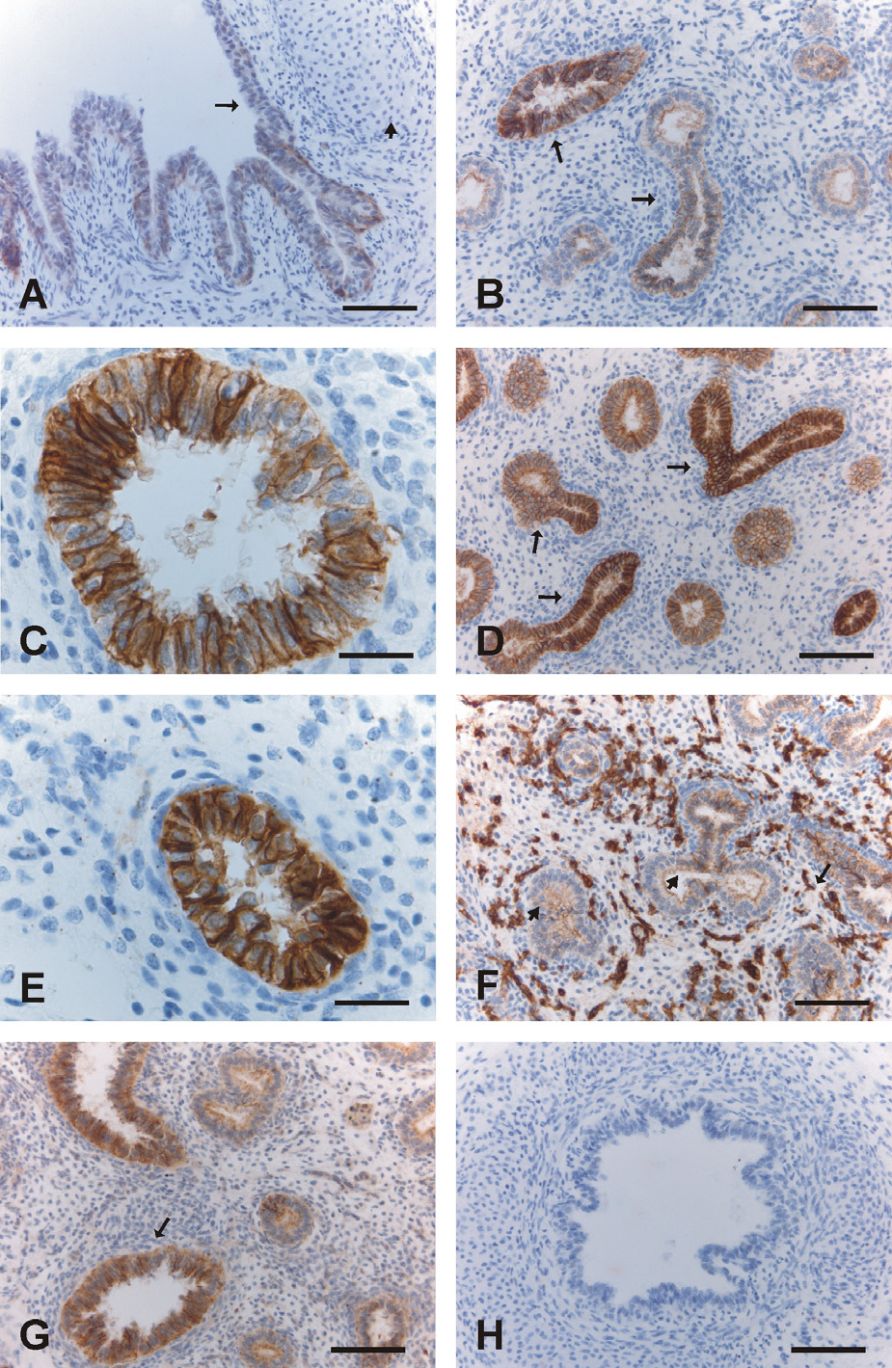
**Immunohistochemical staining for claudins 1, 3, 4, 5 and 7 in pseudoglandular period i.e. in weeks 12-16 in developing human lung**. 1A. Epithelium of a bronchus is positive for claudin-1 (arrow). The short arrow is indicating the cartilage of the bronchus. Scale bar = 80 μm. 1B. Epithelial cells of developing bronchioles are positive for claudin-3 (arrows). Scale bar = 80 μm. 1C. Strong positivity for claudin-3 is seen in the epithelial cells of a bronchiole. Scale bar = 25 μm. 1D-1E. Epithelium of bronchioles is strongly positive for claudin-4 (arrows). Scale bar = 80 μm in Fig 1D, scale bar = 25 μm in Fig 1E. 1F. Endothelial (short arrow) and epithelial cells (arrow) of bronchial epithelium are positive for claudin-5. Scale bar = 80 μm. 1G. Epithelium of bronchi and developing airways are positive for claudin-7 (arrow). Scale bar = 80 μm. 1H. Negative control in which the primary antibody has been substituted with non-immune rabbit mouse serum. Scale bar = 80 μm.

During the pseudoglandular period, the airways are subdividing (i.e. branching morphogenesis). The airways form round gland-like structures, which are lined by pseudostratified epithelia and separated by cellular mesenchymes. Claudin-1 was expressed mainly weakly in bronchi and in some cases also in the larger bronchioles, but it was not detected in small developing airways (Fig 1A). Claudin-3 was expressed strongly in bronchi, and moderately or weakly in bronchioles and small developing airways, which were all positive for claudin-3 (Fig 1B, 1C). Claudin-4 was strongly positive in bronchi, whereas in bronchioles and small developing airways, it was scored as moderate or weak (Fig 1D, 1E). Claudin-5 was strongly positive in endothelial cells, and it was also weakly positive in bronchi, bronchioles and small developing airways (Fig 1F). Claudin-7 was strongly positive in the epithelium of bronchi and bronchioles, and it was also weakly or moderately positive in small developing airways (Fig 1G). During the pseudoglandular period all epithelial cells were positive for claudin-3, -4 and -7 in contrast to claudin-1, which was restricted mainly to the bronchi.

#### Canalicular period (weeks 16 to 28)

**Figure 2:**
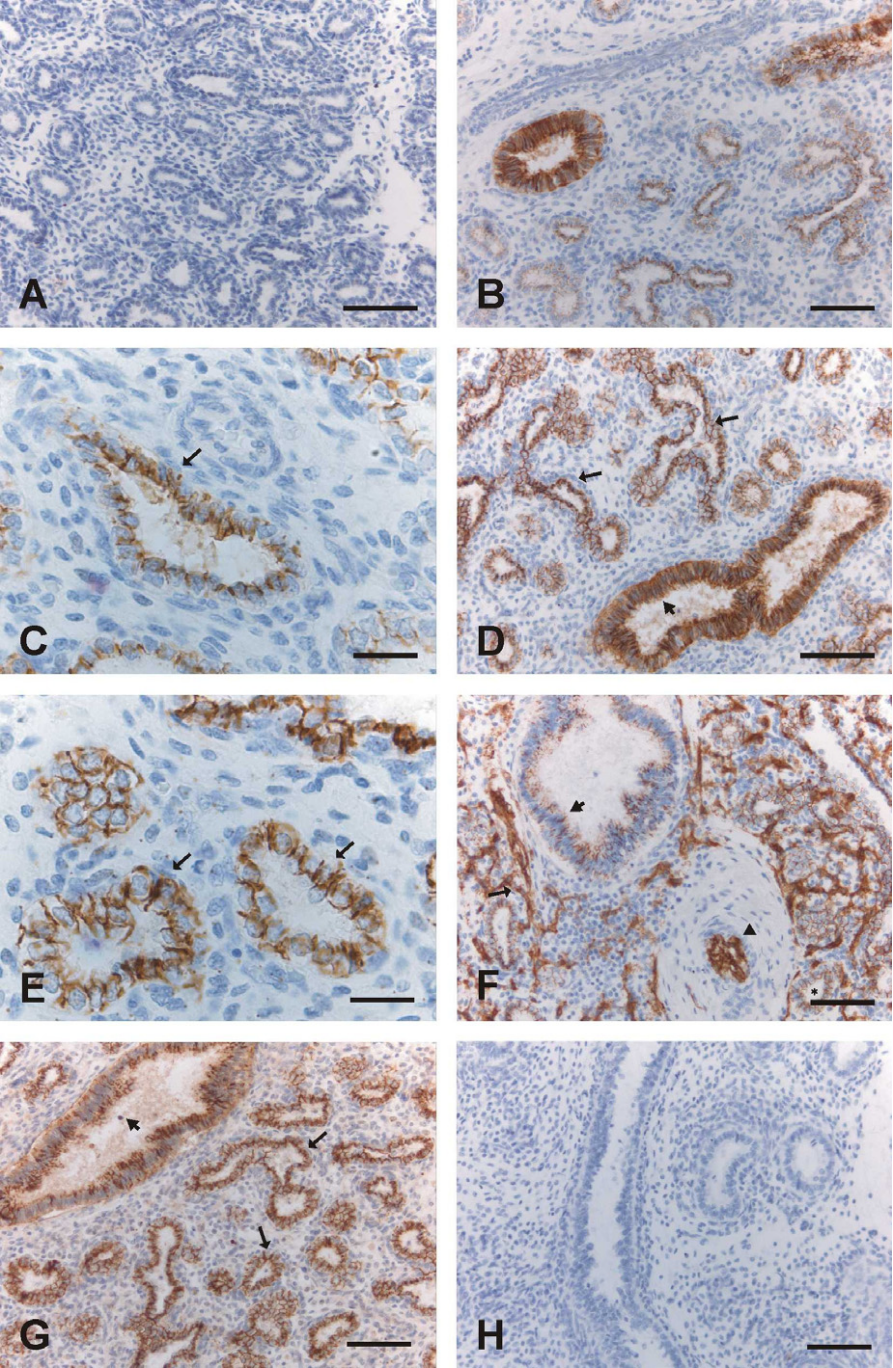
**Immunohistochemical staining for claudins 1, 3, 4, 5 and 7 in the canalicular period i.e. weeks 16-28**. 2A. Epithelial cells including pretype II cells of developing alveoli are negative for claudin-1. Scale bar = 80 μm. 2B. Positive immunoreactivity for claudin-3 is observed in the epithelial cells of a bronchiole (on the left) and in the pretype II cells of developing alveoli. Scale bar = 80 μm. 2C. Pretype II cells of developing alveoli are positive for claudin-3 (arrow). Scale bar = 25 μm. 2D-2E. Epithelium of a bronchiole (short arrow in the Fig 2D) and pretype II cells lining alveoli (arrows in the Figs 2D and 2E) are positive for claudin-4. Scale bar = 80 μm in the Fig 2D, scale bar = 25 μm in the Fig 2E. 2F. Endothelial cells of alveolar capillaries (arrow) and an artery (arrowhead), pretype II cells lining alveoli (asterisk) and epithelium of bronchioles (short arrow) are positive for claudin-5. Scale bar = 80 μm. 2G. Alveolar (arrows) and bronchiolar epithelium (short arrow) are positive for claudin-7. Scale bar = 80 μm. 2H. Negative control in which the primary antibody has been substituted with PBS with haematoxylin counterstain. Scale bar = 80 μm.

The airways are dividing further, the vascular system is developing, and the amount of mesenchyme is decreasing. The epithelium becomes thinner. From week 16, pretype II cells appear, and from weeks 24 to 28, type I and type II pneumocytes start to be detected. The airways are mostly simple and numerous, but in their midst, one can also find branching airways. Claudin-1 was expressed moderately or strongly in the epithelium of bronchi and bronchioles, whereas alveolar epithelium was negative (Fig 2A). Claudin-3, claudin-4 and claudin-7 were expressed positively in alveolar epithelium i.e. in pretype II cells, and the epithelia of bronchi and bronchioles were also strongly positive (Fig 2B, 2C, 2D, 2E, 2G). Claudin-5 was strongly positive in endothelial cells. In addition to the endothelial positivity, it was also weakly positive in epithelium of bronchi and bronchioles, and clearly positive within alveoli in pretype II cells (2F).

#### Saccular (weeks 28 to 36) and alveolar (weeks 36 to 40) periods

**Figure 3:**
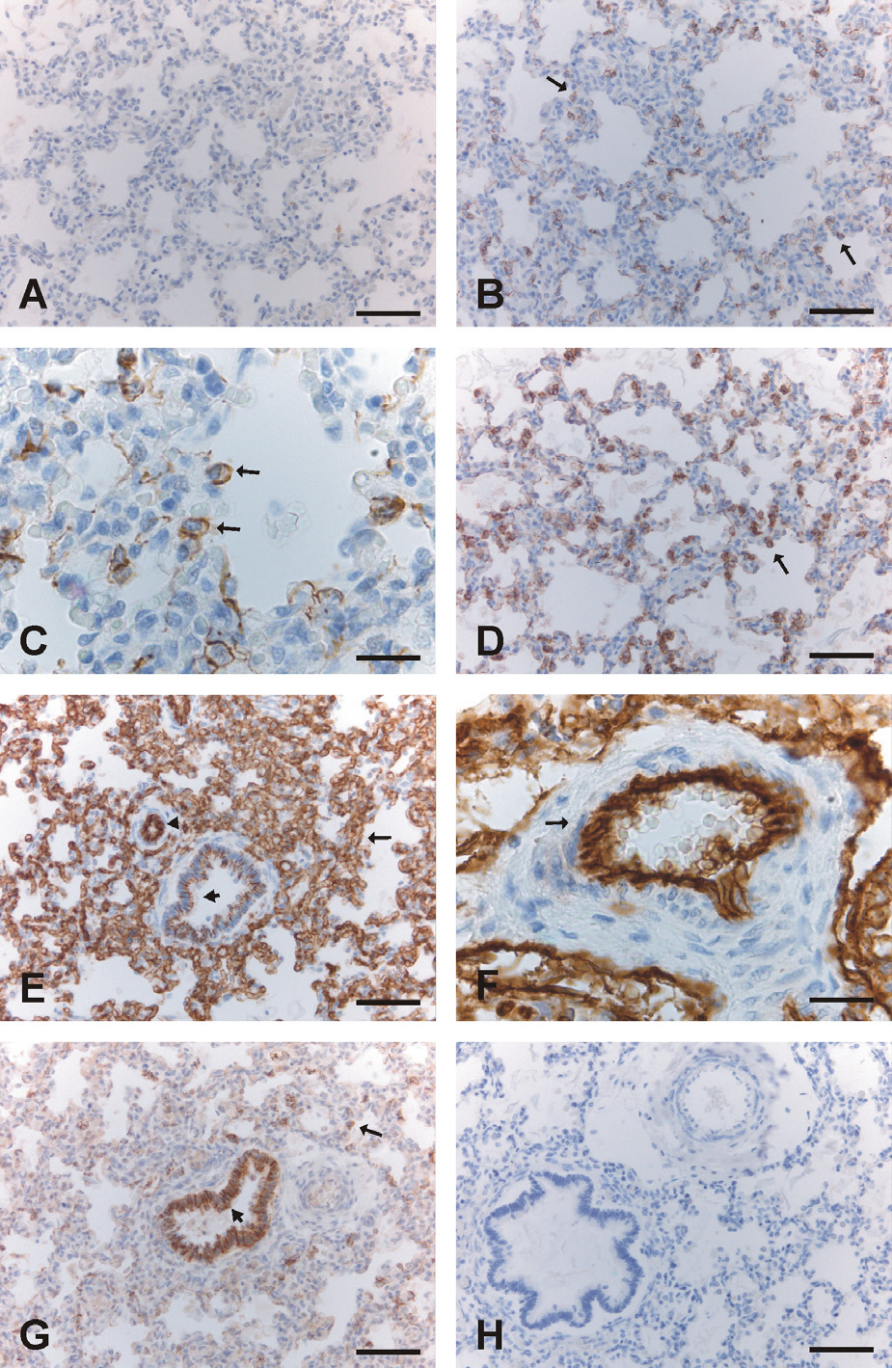
**Immunohistochemical staining for claudins 1, 3, 4, 5 and 7 in the saccular period i.e. weeks 28-36**. 3A. Alveolar epithelium is negative for claudin-1. Scale bar = 80 μm. 3B-C. Type II pneumocytes of developing alveoli are positive for claudin-3 (arrows). Scale bar = 80 μm in the Fig 1B, scale bar = 25 μm in the Fig 1C. 3D. Type II pneumocytes lining alveoli are positive for claudin-4 (arrow). Scale bar = 80 μm. 3E. Endothelial cells of capillaries (arrow) and an artery (arrowhead), and also epithelium of a bronchiole (short arrow) are positive for claudin-5. Scale bar = 80 μm. 3F. Strong immunoreactivity for claudin-5 is seen in endothelial cells of an artery (arrow). Scale bar = 25 μm. 3G. Type II pneumocytes of alveoli (arrow) and epithelial cells of a bronchiole (short arrow) are expressing claudin-7. Scale bar = 80 μm. 3 H: Negative control in which the primary antibody has been substituted with non-immune rabbit mouse serum. Scale bar = 80 μm.

**Figure 4:**
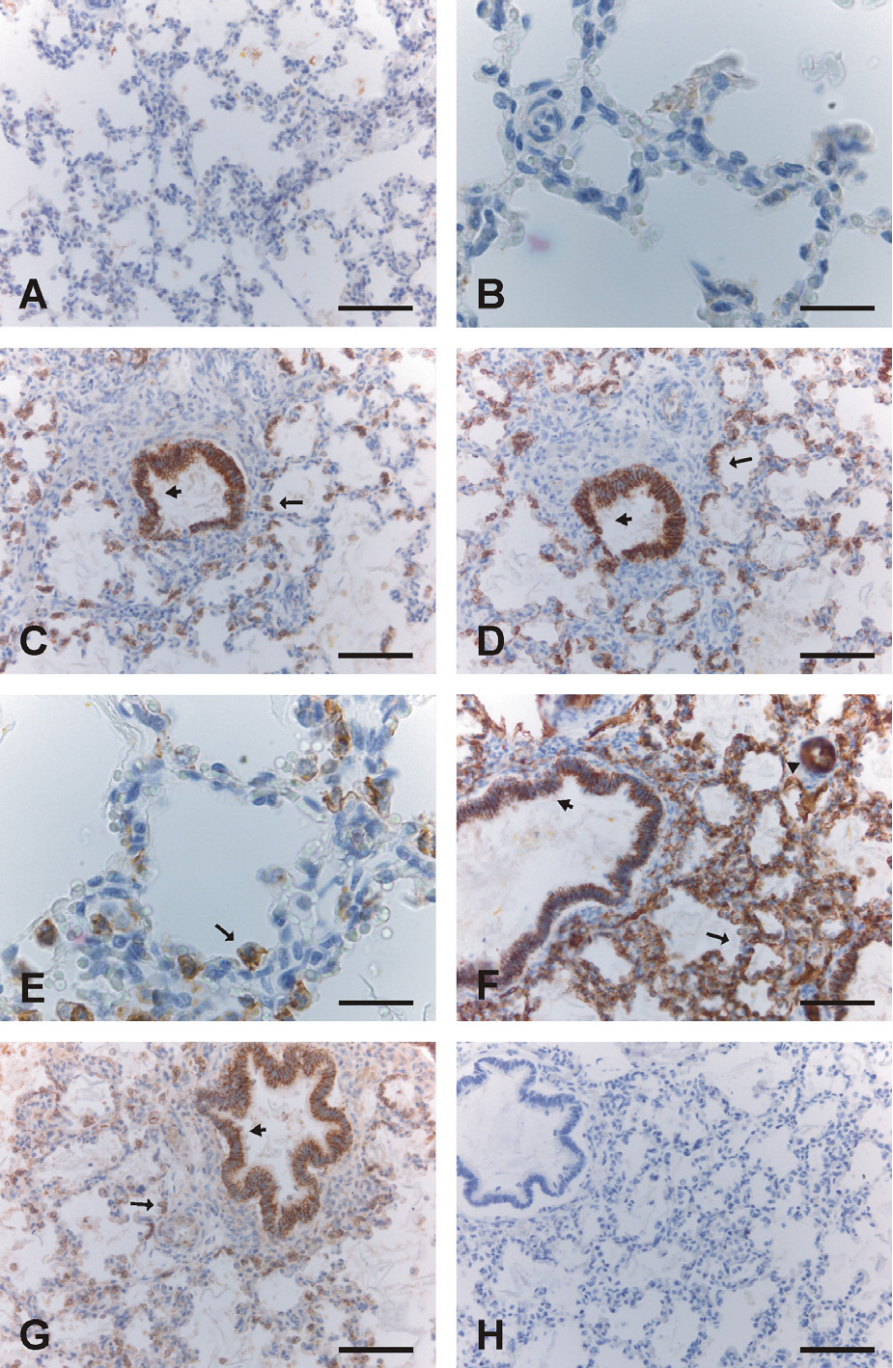
**Immunohistochemical staining for claudins 1, 3, 4, 5 and 7 in the alveolar period i.e. weeks 36-40**. 4A-4B. Epithelium of alveoli is negative for claudin-1. Scale bar = 80 μm in the Fig 4A, scale bar = 25 μm in the Fig 4B. 4C, 4D, 4E: Type II pneumocytes of alveoli (arrows) and epithelium of bronchioles (short arrows) are positive for claudin-3 (4C) and claudin-4 (4D, 4E). Scale bar = 80 μm in the Figs 4C and 4 D, scale bar = 25 μm in the Fig 4E. 4F. Endothelial cells of alveolar capillaries (arrow) and an artery (arrowhead), and bronchiolar cells (short arrow) are positive for claudin-5, whereas alveolar epithelium is not. Scale bar = 80 μm. 4G: Epithelium of a bronchiole (short arrow) and type II pneumocytes of alveoli (arrow) are positive for claudin-7. Scale bar = 80 μm. 4H. Negative control in which the primary antibody has been substituted with PBS with haematoxylin counterstain. Scale bar = 80 μm.

The saccular and alveolar periods are characterized by an increase in the gas-exchanging surface area, and a decrease of the mesenchymes between saccules. Small crests appear in the walls of sacculi, ultimately developing into alveoli from week 36 onward. Claudin-3, -4 and -7, but not claudin-1 and -5, were positive in type II pneumocytes (Fig 3A-3G, 4A-4G). Claudin-1, -3, -4 and -7 were expressed strongly in bronchi and bronchioles (Fig 3G, 4C, 4D, 4G). Claudin-5 positivity was strong in endothelial cells of arteries, capillaries, veins and lymphatic vessels, whereas bronchial epithelium displayed faint positivity and alveolar epithelium remained negative (Fig 3F, 4F).

Mesothelium, fibroblasts, myofibroblasts, smooth muscle cells and chondrocytes were negative for all of the claudins studied. In some cases during the pseudoglandular period, trachea was included within the tissue sample, and its epithelium was positive for all claudin studied.

The results of the scoring of the immunoreactivity of various claudins are shown in Table 2. The exact scores of each case for each claudin in various periods are represented.

**Table 2:** Immunoreactivity scores for claudin-1, -3, -4, -5 and-7 in different types of pulmonary cells in developing human lung during various gestational periods.

Claudin type	Pseudo-glandular period day 52-week 16 13 cases (scores/number of cases)	Canalicular period weeks 16-28 17 cases (scores/number of cases)	Saccular period weeks 28-36 9 cases (scores/number of cases)	Alveolar period weeks 36-40 8 cases (scores/number of cases)
**Claudin-1**				
bronchus	+/10, ++/3	+++/17	++/8, +++/1	++/1, +++/7
bronchioles		+/5, ++/12	+/8, ++/1	++/5, +++/3
- large	+/13			
- small	negative/13			
alveolus	# negative/13	negative/17	negative/9	negative/8
endothelial cell	negative/13	negative/17	negative/9	negative/8

**Claudin-3**				
bronchus	+++/13	+++/17	+++/9	+++/8
bronchiolus	++/6, +++/7	+++/17	+++/9	+++/8
alveolus	# +/4, ++/9	* +/8, ++/9	& +/7, ++/2	& +/7,++/1
endothelial cell	negative/13	negative/17	negative/9	negative/8

**Claudin-4**				
bronchus	+++/13	+++/17	+++/9	+++/8
bronchiolus	+++/13	+++/17	+++/9	+++/8
alveolus	# +/3, ++/10	* +/4, ++/13	& +/5 ++/4	& +/2 ++/6
endothelial cell	negative/13	negative/17	negative/9	negative/8

**Claudin-5**				
bronchus	+/13	+/17	+/9	+/8
bronchiolus	+/13	+/17	+/9	+/8
alveolus	# +/13	* +/17	negative/9	negative/8
endothelial cell	+++/13	+++/17	+++/9	+++/8

**Claudin-7**				
bronchus	+++/13	+++/17	+++/9	+++/8
bronchiolus	++/3, +++/10	+++/17	+++/9	+++/8
alveolus	# +/13	* +/7,++/9, +++/1	& +/8, ++/1	& +/8
endothelial cell	negative/13	negative/17	negative/9	negative/8

# = small developing airways, not alveoli

* = pretype II cells

& = type II pneumocytes

##### RT-PCR

Total RNA was isolated from paraffin embedded tissues and converted to cDNA. Relative quantity of claudins 1, 3 and 4 were studied by RT-PCR. The results of the RT-PCR assays demonstrated that it was possible to isolate RNA fragments large enough for amplification from each sample. Minor differences could be detected between the samples in the RNA amount of GAPDH that can be considered as a house-keeping gene as there were no statistically significant differences between various gestational stages.

**Figure 5:**
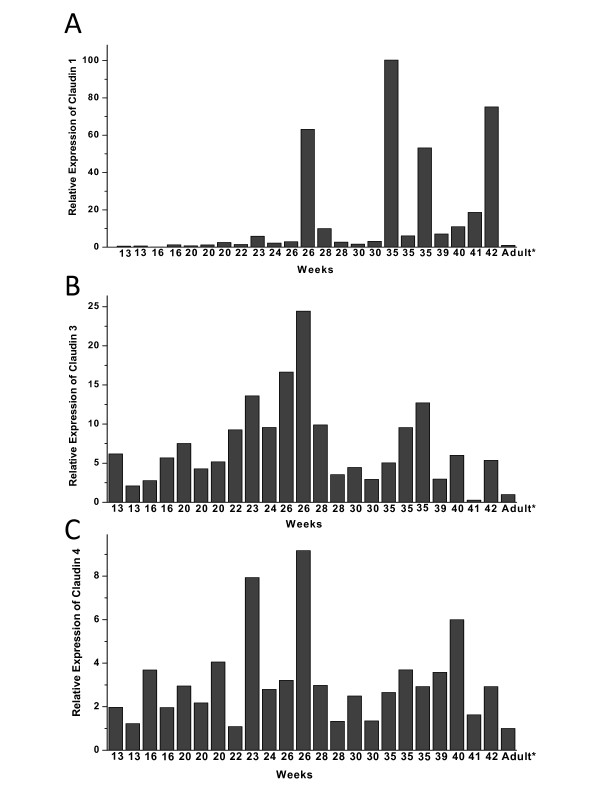
**Quantitative RT-PCR analysis of claudin-1 (5A), claudin-3 (5B) and claudin-4 (5C) expression of RNA isolated from paraffin embedded lung tissue samples**. Each bar represents a single lung sample from weeks 13 to 42 related to adult lung (value = 1). The analyses show a great individual variability in the expression of all claudins analysed. Expression of claudin-1 RNA increases towards the end of gestation, whereas RNA expressions of claudin-3 and -4 are at their highest in the canalicular period.

**Figure 6:**
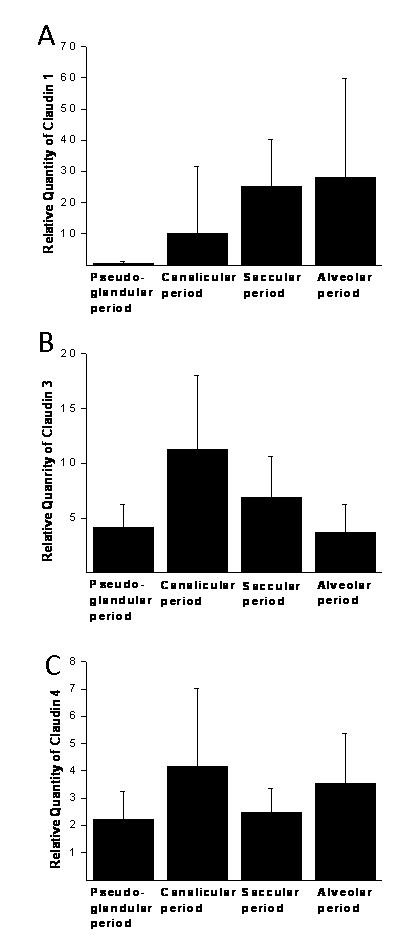
**Summary of the quantitative RT-PCR results showing the expression of claudin-1 (A), claudin-3 (B) and claudin-4 (C) in various gestational stages**. Results are shown as mean values and error bar represents the standard deviation.

Quantity of each claudin was related separately to adult lung sample (value = 1) and therefore the values are relative and the values of different claudins are not comparable. For example the values of claudin-1 can be compared only to other values of claudin-1, but not to claudin-3 or -4. Variable amount of RNAs of claudin-1 (Fig 5A), claudin-3 (Fig 5B) and claudin-4 (Fig 5C) were detected. Claudin-1 RNA level was low (mean 0.60, SD 0.47) at week 12 to week 16 increasing (p = 0.016) slightly after week 20 (mean 9.98, SD 21.55) (Fig 6). The expression of claudin-1 RNA was further increased during saccular (mean 25.25, SD 37.81) and alveolar periods (mean 27.95, SD 31.82). RNA-expression of claudin-3 was low during pseudoglandular period (mean 4.18, SD 2.05) and increased (p = 0.048) towards the canalicular period (mean 11.31, SD 6.70) after which it decreased in saccular (mean 3.65, SD 2.61) and alveolar (mean 3.65, SD 2.61) periods. RNA level of claudin-4 appeared to increase from pseudoglandular period (mean 2.21, SD 1.04) to canalicular period (mean 4.17, SD 2.85) when its level was at its highest. Subsequently, expression of claudin-4 RNA was observed to decline in saccular period (Mean 2.49, SD 0.87) and it then appeared to stay at that level until week 42 (mean 3.53, SD 1.83). Amounts of RNAs of claudins 1, 3 and 4 were slightly higher in the end of alveolar period of developing lung than in healthy adult human lung.

## Discussion

This is the first study to demonstrate the expression and cell-specific localization of claudin-1, -3, -4, -5 and -7 in developing human lung at the tissue level. We observed that the expression of claudin-1 was more restricted compared to the other claudins studied, since it was positive only in the epithelium of bronchi and bronchioles, but not in the alveolar epithelium. In a previous study by Wang et al on developing rat lung tissue claudin-3, -4 and -5 were co-expressed in type II alveolar epithelial cells, but only a trace amount of claudin-1 could be detected in these cells, a finding which is in line with our present study [24]. Similar to our findings claudin-5 was expressed throughout the alveolus [13], although our study clarified that the expression of claudin-5 was due to its presence in alveolar endothelial cells. The study of Daugherty and co-authors indicated that fetal lung cells cultured in a medium containing elements which promoted alveolar epithelial cell differentiation to a type II phenotype expressed claudin-1, -3-, -4, -5 and -7 [25]. This finding is in concordance with ours except for claudin-1. The difference may be due to different in vitro methodology using experimentally manipulated fetal lung cells. We also observed a distinct expression profile of claudins in bronchial cells. Claudin-1 was expressed later in gestation i.e. it was negative in the small sized developing airways during the pseudo-glandular period. In contrast these airways were clearly positive for claudin-3, -4 and -7 and faintly positive for claudin-5 during that period. The most abundant expression of claudins by immunohistochemistry was observed during the canalicular period from week 16 to week 28, when alveoli and acini are developing. The cells that line the alveoli during most of that period are pretype II pneumocytes, which are developing into type II pneumocytes and type I pneumocytes during weeks 24-28. Claudin-3, -4, -5 and -7 were positive in pretype II cells during the canalicular period, while later during the saccular and alveolar phases from week 28 to week 40 only claudin-3, -4 and -7 were present in type II pneumocytes, and claudin-5 was not expressed any longer in these cells.

During the pseudoglandular period when terminal bronchioli are developing, claudin-3, -4 and -7 were positive in all epithelial cell-lining airways, but claudin-1 was positive mainly in large bronchi and its expression was usually quite weak. Claudin-3, -4, -5 and -7 were positive and claudin-1 negative in pretype II cells of alveoli during the canalicular period indicating that claudin-3, -4 and -7, but not claudin-1, are related to the development of acini and the differentiation of alveolar epithelial cells. In our previous study with samples from normal human adult lung, sarcoidosis and idiopathic pulmonary fibrosis (IPF) claudin-3, -4 and -7, but not claudin-1, were positive in normal type II pneumocytes, a finding which is in agreement with the results of the present study [26]. Claudin-1 was, however, detected in the metaplastic alveolar epithelium of IPF and sarcoidosis. Claudin-5 was mainly positive in the endothelial cells of adult human lung, sarcoidosis and IPF, but it was also faintly positive in the bronchial and metaplastic alveolar epithelium of the diseased lung, a finding which is confirmed by the results of the present study demonstrating epithelial expression in the developing bronchial epithelium and in the alveoli during the canalicular period.

In our previous studies with a similar material, we investigated the expression of tenascin-C, collagen I and collagen III mRNAs in developing human lung [4,5,27]. Some mRNAs are stable for 24 hours under post-mortem conditions, allowing *in situ *hybridization to be performed [28]. In the present study, the results of immunohistochemistry were confirmed by RT-PCR. This method detected measurable amounts of claudin-1, claudin-3 and claudin-4 mRNAs in all 23 cases studied. The amount of claudin-1 mRNA was low during the pseudo-glandular period, a finding which is concurrent with the results of immunohistochemistry. Furthermore, the mRNAs of both claudin-3 and claudin-4 were at their highest during the canalicular period, a finding which is also in concordance with the results obtained by immunohistochemistry, since during the canalicular period, the expressions of claudin-3 and -4 were positive both in bronchioles and alveoli. During the canalicular period all pretype II cells were positive for claudin-3 and -4, but negative for claudin-1. The decrease in the amounts of the mRNAs coding for claudin-3 and -4 after the canalicular period is attributable to the development of type I and type II pneumocytes during the saccular and alveolar periods since type I pneumocytes are negative for claudin-3 and -4. With the prospective procedure and a standardized fixation time, it might be possible to achieve even more accurate information about the amounts of mRNA of different claudins.

We conclude that claudin-1, -3, -4, -5, and -7 are expressed in developing human lung from week 12 to week 40 with distinct locations and in divergent quantities reflecting changes in cellular polarity and permeability during different phases of epithelial development. The expression of claudin-1 is restricted to the bronchial epithelium, whereas claudin-3, -4 and -7 are positive also in alveolar epithelium as well as in bronchial epithelium suggesting that these claudins in contrast to claudin-1 are also involved in the development of acinus and the differentiation of alveolar epithelial cells. Claudin-5 is localized mainly in endothelial cells but its expression in pretype II alveolar epithelium indicates that it may be involved in the development of acinar epithelial cells.

## Competing interests

The authors declare that they have no competing interests.

## Authors' contributions

RK participated in the design of the study, collected the study material, analyzed the immunohistochemical and clinical data of the patients, and drafted the manuscript. HM participated in the study by carrying out the quantitative RT-PCR analysis, created the RT-PCR figures and helped to draft the manuscript. SL participated in design of the study, design and analysis of RT-PCR-analyses and helped to draft the manuscript. TH participated in design of the study, design of RT-PCR-analyses and helped to draft the manuscript. YL participated in design of the study and helped to draft the manuscript. All authors has read and approved the final manuscript
